# Checklist Model to Improve Work Practices in Small-Scale Demolition Operations with Silica Dust Exposures

**DOI:** 10.3390/ijerph9020343

**Published:** 2012-01-24

**Authors:** Custodio Muianga, Carol Rice, Thomas Lentz, James Lockey, Richard Niemeier, Paul Succop

**Affiliations:** 1 Department of Environmental Health, College of Medicine University of Cincinnati, 3223 Eden Ave., Kettering Laboratory, Cincinnati, OH 45267, USA; Email: ricech@ucmail.uc.edu (C.R.); lockeyje@ucmail.uc.edu (J.L.); succoppa@uc.edu (P.S.); 2 Center for Industrial Studies, Safety and Environment, Eduardo Mondlane University, P.O. Box 257, Maputo, Mozambique; 3 Education and Information Division, National Institute for Occupational Safety and Health (NIOSH), CDC, 4676 Columbia Parkway, Cincinnati, OH 45226, USA; Email: tbl7@cdc.gov (T.L.); rwn1@cdc.gov (R.N.)

**Keywords:** work practices, construction sector, dust exposure controls, Mozambique

## Abstract

A systematic approach was developed to review, revise and adapt existing exposure control guidance used in developed countries for use in developing countries. One-page employee and multiple-page supervisor guidance sheets were adapted from existing documents using a logic framework and workers were trained to use the information to improve work practices. Interactive, hands-on training was delivered to 26 workers at five small-scale demolition projects in Maputo City, Mozambique, and evaluated. A pre-and-post walkthrough survey used by trained observers documented work practice changes. Worker feedback indicated that the training was effective and useful. Workers acquired knowledge (84% increase, *p* < 0.01) and applied the work practice guidance. The difference of proportions between use of work practice components before and after the intervention was statistically significant (*p* < 0.05). Changes in work practices following training included preplanning, use of wet methods and natural ventilation and end-of-task review. Respirable dust measurements indicated a reduction in exposure following training. Consistency in observer ratings and observations support the reliability and validity of the instruments. This approach demonstrated the short-term benefit of training in changing work practices; follow-up is required to determine the long-term impact on changes in work practices, and to evaluate the need for refresher training.

## 1. Introduction

The construction industry represents 5 to 15% of the economy of most developing countries and is generally among the three industries with highest work-related injuries and diseases. An important part of construction operations is demolition [1], where the workforce is often from the informal sector; small-scale, self-employed contractors, and trade-related unskilled workers (including children less than 15 years old) often work at these temporary worksites. These small-scale operations involve physically demanding work and represent potential for exposure to many kinds of dusts, including crystalline silica, stone and concrete-based building materials. These demolition tasks include breaking up, dismantling, chipping, cutting, hammering, crushing, loading, hauling, dumping, and dry sweeping [2]. Exposure to respirable crystalline silica dust (*i.e.*, generally the mineral quartz and designated in this article as silica dust) is known to cause silicosis, and may be associated with increased risk of lung cancer, pulmonary tuberculosis, chronic obstructive pulmonary disease, autoimmune disease and renal diseases [3]. 

In developed countries, progress has been made in reducing silica exposures through the implementation of engineering and other controls and enforcement of regulations to limit exposure [1,2,3,4]. These efforts are built on a long history of silica measurement and disease surveillance. 

In Africa and Eastern countries, only South Africa has routinely measured silica exposures during the past century and implemented controls. Reports from South Africa provide insights to the continuing risk of tuberculosis and nontuberculous mycobacteria-related diseases among silica-exposed workers [5,6,7]. A recent review of tuberculosis and silicosis found that the risk of a patient with silicosis developing tuberculosis is in the range of 2.8 to 39 times higher than that found for healthy controls [8]. A study among South African gold miners showed that the relation between silica and tuberculosis was compounded by the human immunodeficiency virus (HIV) [9]. These added risks to silica-exposed individuals are of great concern in industrializing countries. Silicosis is not often diagnosed in developing countries due to the lack of qualified professionals to determine an occupational history of silica exposure and identify the characteristic radiological features and exclude other conditions, TB is more consistently diagnosed and the estimated prevalence rate of TB is 504 per 100,000 population; similarly, HIV is now diagnosed and the estimated prevalence rate of HIV/Acquired Immune Deficiency Syndrome (AIDS) is 16.2 per hundred thousand among the population aged 15 to 49 years [10,11]. Any effort to reduce silica dust exposures is an important public health strategy to reduce this public health burden [6,7].

Exposure control strategies for small-scale construction activities such as demolition are challenging due to the short-duration of work at a site. Short checklists, control guides and information sheets are available in developed countries to assist workers and managers in identifying sources of exposure and necessary controls. In the U.S., the CPWR—The Center for Construction Research and Training—developed a task-based exposure assessment model in which the task was viewed as a primary building block of an exposure assessment framework [12,13]. A joint research project of the US Mount Sinai Construction Hygiene and Ergonomics Program (CHEP), and Hunter College, Urban Public Health Program resulted in the Blueprint Guides for managing silica control programs in construction. Each guide included step-by-step instructions for planning, implementing and evaluating key program components. The guides contained explanatory notes and hyperlinks to checklists, sample forms, and websites containing useful information related to managing a lead and/or silica health hazard control program [14]. In the United Kingdom (UK), the Health and Safety Executive (HSE) developed a free internet tool for identifying good control practices [15]. Partially as a result of this experience, the European Union (EU) multi-sector Negotiation Platform on Silica (NEPSI) developed a Good Practice Guide and detailed task control guidance sheets [16]. These toolkits are widely used in the UK and Europe [17]. 

These products have been useful guidance in developed countries, but may not be immediately transferable to workers in other countries. Our previous research on silica dust exposure control strategies in small-scale concrete and masonry demolition operations showed limited usability of the UK HSE Control of Substances Hazardous to Health (COSHH) Silica Essentials, and the NEPSI Good Practice Guide in Mozambique due to a mis-match between the level of engineering controls recommended and local availability Purchase, transport, set up and maintenance costs of these engineering technologies cannot be afforded by small-scale demolition contractors in Mozambique. Other constraints in the use of these guidance tools included language barriers (English not native language in industrializing nations), lack of availability of communication technologies (Internet access), and extensive text and technical information on both hazard and controls may limit their use by workers and some supervisors due to lack of time to study the material and the high level of literacy required for use [18]. While a valuable resource to address silica hazards prevention and controls, the existing tools needed adaptation to working conditions in developing countries [17,18].

The work reported here addresses these gaps. A systematic approach was developed to review control guidance used in developed countries, and adapted for use in Mozambique. A logic framework and step-by-step description of the development, results of its implementation and evaluation at five construction projects with small-scale demolition activities in Maputo City, Mozambique, Africa are presented. 

## 2. Methods and Materials

### 2.1. Steps for Developing Exposure Control Guidance

**Figure 1:**
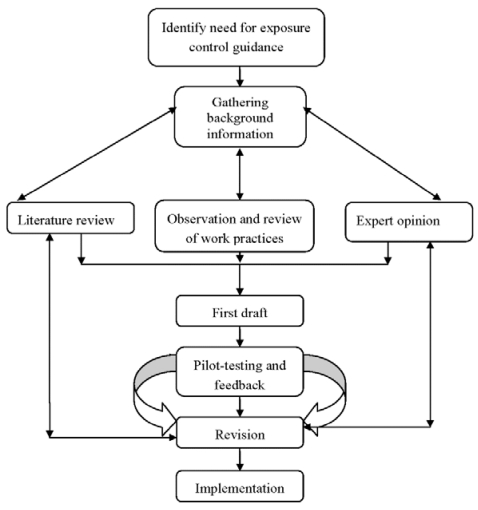
Logic framework for checklist development.

A systematic and iterative approach was used, including: (i) identifying need; (ii) gathering background information; (iii) writing first draft; (iv) pilot-testing for feedback; (v) revising, and (vi) implementing. Figure 1 presents the framework of the model. The description of each step is presented below.

#### 2.1.1. Identifying Need

The need for exposure control guidance can be based on a preliminary evaluation of workplace conditions observed during a walk-through and talking with workers and supervisors. Documentation of exposure from air sampling or from disease surveillance programs may also document a need. Exposure control guidance matched to the work organization is most likely to be implemented [18].

#### 2.1.2. Gathering Background Information

The background information search had three components, including literature review, observations and review of work practices, and expert advice to acquire innovative, unpublished approaches to exposure control in small-scale demolition operations. The literature review focused on practical, available and regionally accessible exposure controls, self-efficacy (how workers involve themselves to reduce exposures) and guidance content. These included the UK HSE COSHH Essentials, NEPSI good practice guide, the International Chemical Toolkit from the International Labor Organization (ILO), the Blueprint Guide for managing silica control program in construction developed by Clark *et al.* [14,15,16,17,18]. Work practices were observed during small building renovation and remodeling projects in a university setting in Mozambique and documented with photographs, published articles and hazard evaluation reports of small-scale demolition operations were reviewed [2,3,12,13]. Experts were asked to provide input regarding work practice observations, and feasible approaches to exposure control. 

#### 2.1.3. Writing First Draft

The core of exposure control guidance is a checklist that can be used by workers to routinely implement actions. The initial version of the checklist was developed based on the guidelines for checklist development presented by Stufflebeam [19], a checklist for the construction industry by the Montana Department of Labor and Industry [20], and the results from the reviewed good practice guidance strategies.

#### 2.1.4. Pilot-Testing and Feedback

The checklist draft was pre-tested with professionals at a local meeting of occupational hygiene professionals, followed by construction workers at a national meeting and finally with demolition workers and supervisors. 

#### 2.1.5. Revision(s)

Each pilot-test resulted in revision, and the revision was presented to the next group. 

#### 2.1.6. Implementation

Implementation and evaluation required training workers. The training development process included the following components: identifying goals and objectives, developing learning activities, conducting the training, evaluating program effectiveness, and improving the program. [21]. Content included the good work practice components covered in the control guidance checklist. Other factors included describing the target group, training agenda, appropriate facilities, facilitator preparation, and necessary audio visual aids.

The training material was pilot tested with construction workers (supervisors and employees), construction safety professionals, and professionals with experience in both worker training in the construction sector and small-scale business. Comments and critiques were used to revise the materials.

### 2.2. Steps for Evaluation of the Control Guidance

The use of the guidance was based on observations made during a walkthrough survey. In order to standardize this process, the following steps were undertaken: (i) development of walkthrough survey guide; (ii) training data collectors/observers; (iii) pretraining assessment of work practices; (iv) worker training and (v) posttraining assessment of work practices. 

#### 2.2.1. Development of Walkthrough Guide to Assess Control Guidance Use

The walkthrough survey guide was developed following the American Industrial Hygiene Association (AIHA) strategy for assessing and managing occupational exposures [22] and the occupational hygiene survey and audit protocols developed by Orr and Labato [23]. The walkthrough survey guide was composed of work practices presented on the checklists plus space to describe the worksite, and list equipment, tools and material observed in use by the demolition crew during each of the demolition tasks. 

#### 2.2.2. Training of Observers/Data Collectors

In order to control for information and observation bias, two observers were trained with the guide to observe and record work practices. The observers were trained and calibrated by one author (CM) to record accurate observations prior to the field work. During pretraining and posttraining assessments, the independent observations of the two data collectors were compared at the end of each shift to identify any inconsistencies.

#### 2.2.3. Pretraining Assessment of Work Practices

The two trained data collectors recorded observed work practices during demolition tasks. At least two random checks in each work shift were performed and comparison of the results was done at the end of the shift. Interobserver agreement was evaluated. 

#### 2.2.4. Worker Training and Target Population

A task-based, interactive hands-on training program was developed. The total duration of worker training was six hours with two-and-one-half h for supervisor training and three-and-one-half h for a combined employees and supervisors training session. The supervisor training was based on structured dialogue in a round-table setting near the demolition worksite. The supervisors were challenged to explain the process, tools and materials used and work practices. This discussion was followed by a field demonstration. The training for employees and supervisors included: (i) silica dust health hazard information; (ii) preplanning and procedures for applying good work practice controls while performing each task and (iii) step-by-step small-scale demolition processes and reinforcement of key points. Photographs of typical tasks of small-scale concrete and masonry demolition operations in Mozambique and a small-scale concrete and hands-on demolition simulation exercise were used.

The seven levels of evaluation of worker training provide a complete assessment of the program including the checklist, its implementation and short-term use. These levels include: (i) tracking attendance; (ii) curriculum content, methods, and delivery; (iii) satisfaction and opinion of the trainees; (iv) knowledge acquisition and understanding; (v) skills acquisition; (vi) transfer of learning to the workplace; and (vii) the impact of the training. Parry and Berdie presents a detailed description of each level of training evaluation [24]. The feedback and participant satisfaction included Likert-scale items on content and quality of delivery, facilitation, training effectiveness and usefulness of work practices and three open ended questions.

#### 2.2.5. Posttraining Assessment of Work Practices

After training, the workers performed their routine work. The postintervention evaluation was done through observation of work practices. Descriptive statistics were computed. As appropriate, statistical significance was determined using the two proportions *Z*-test and McNemar’s Test [25].

## 3. Results

### 3.1. Steps for Developing Exposure Control Guidance

#### 3.1.1. Identifying Need

Previous work documented dusty conditions (surface deposits and visible dust clouds) during small-scale demolition operations at several different university worksites. Workers performed demolition tasks without any type of dust exposure preventive measures, and both construction employees and supervisors reported dust exposures as part of their normal working conditions [18]. The alpha quartz content in settled dust bulk samples from construction materials collected in five demolition projects ranged from 18 to 40%, arithmetic mean of 30%. Thus it was likely that these workers were exposed to silica and methods to reduce exposure were needed. 

#### 3.1.2. Background Information

Observation and review of work practices revealed that demolition operations were conducted both indoors and outdoors and involved only manual labor. Recommended exposure controls in the reviewed work practice guidance approaches included dust extraction with local exhaust, use of control cab equipped with a HEPA filtration system, water dust suppression system, and personal protective equipment (PPE) including respiratory protection. The control guidance sheets were available through internet systems, but with limited accessibility in Mozambique. These control guidance sheets were in English and included extensive text and technical information on both hazard and controls 

Manual tools included sledge hammers, mason hammers, chisels and scrapers, brooms, sweepers, and shovels. Electrically or pneumatically driven jackhammers and heavy-duty vacuum cleaners were not available locally in Mozambique.

By observation in Mozambique, the process of small-scale manual demolition was composed of the following steps: (i) marking a diagram of the area to be demolished; (ii) breaking-up concrete and masonry using nonpowered hand tools such as sledge hammers, hammers, chisels, scrapers; (iii) concentrating (bringing together) demolition debris using tools such as shovels, brooms or sweepers, hands, and sheets to carry material; (iv) clearing-up (removing or transferring from surface to a container, and transporting) debris from the demolition site to designated salvage or disposal point using tools such as shovels, brooms, sweepers, hands, metallic or plastic can/bucket, sack, wheelbarrow or hand-truck. The sequence of the tasks depended on various factors such as the number of people assigned (usually less than ten people), workspace, tools available for the work, and activities following demolition (renovation or new construction).

Personal contact with experienced professionals in the field working in institutions such as NIOSH, CPWR, the Institute for Risk Assessment Science (IRAS) in the Netherlands, and the UK HSE showed that many construction work activities included similar tasks, regardless of location. It was recommended that new guidance sheets should be short and use text written in plain language with white space. 

#### 3.1.3. Writing First Draft

The first draft of the checklist was restricted to housekeeping and cleaning and was composed of four sections and was four pages in length. The first three sections included questions on exposure-reduction and the fourth section included items common to all demolition tasks. 

#### 3.1.4. Pilot-Testing and Feedback

Professionals provided feedback on structure, content and usefulness. Recommendations included using a separate checklist for each task, replace open-ended questions with statements requiring yes/no answers, combining guidance and checklist in one page and addition of space for alternative exposure controls. This iterative process of pilot, feedback, and revision was repeated three times and involved all stakeholders.

#### 3.1.5. Revision(s)

In response to feedback, an adaptation of UK HSE COSHH Silica Essentials guidance sheets format was used and a fourth checklist for cleaning up debris and soil was added. Changes made after the national occupational health and safety meeting included formatting each task-based checklist and broadening the content to cover other activities involved in the process of small-scale demolition operations. Health hazard information was added. The exposure reduction strategy in the second draft of each task-based checklist was based on the good work practice components: (i) pre-planning; (ii) use of water to suppress dust; (iii) use of a high efficiency particulate air (HEPA) filtered vacuum to remove dust; (iv) use of natural ventilation and general ventilation; (v) availability and practice of basic hygiene and sanitation; (vi) availability and usage of basic personal protective equipment (PPE); and (vii) checklist review after task completion.

Additional recommended changes from construction professionals participating in the national conference included the development of two types of task-based checklists: a one-page checklist for employees, a multiple-page document for supervisors or managers. The one-page checklist included sufficient information for an employee to understand silica dust health hazards and how to reduce silica dust exposures using good work practices while performing the task. The supervisor document contained the one-page employee checklist and information on: (i) why one should be concerned about silica dust exposures (regulation compliance requirements, health hazard information and business and economic point of view); (ii) access and premises; (iii) design and equipment; (iv) maintenance; (v) examination and testing; (vi) personal protective equipment; (vii) cleaning, housekeeping and personal hygiene practice; (viii) training and supervision; (ix) further information on control methods for silica dust exposures and useful links. This shorter, multiple-page document follows the general framework used in the COSHH Silica Essentials and the NEPSI good work practice guide except it presents specific information on Mozambican regulations.

**Figure 2:**
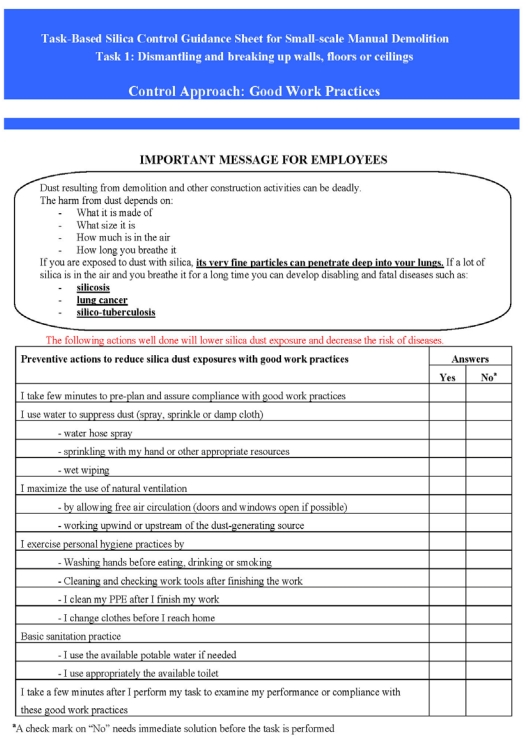
Task-based checklist for dismantling and breaking up concrete and masonry structures.

Input from workers in Mozambique was sought. Based on their input, only three tasks were needed: (i) breaking concrete and masonry structures; (ii) cleaning, concentration and removal of demolition debris and scraps; and (iii) transferring and transporting the debris or scraps for salvage and disposal. Therefore, the fourth checklist on cleanup was eliminated. Figure 2 illustrates the final one page employee checklist for Task 1: breaking concrete and masonry structures for workers.

#### 3.1.6. Implementation

Four supervisors and 22 employees from two demolition crews doing renovations at five sites at a university setting in Maputo City, Mozambique participated. IRB approval was obtained from the University of Cincinnati, OH, USA and the Eduardo Mondlane University, Mozambique. Crew 1 was composed of four supervisors, four masons and six assistant masons employed by the university. Their daily duties included renovations, remodeling and maintenance. Their construction projects always involved some demolition operations of concrete and masonry structures. Crew 2 was composed of two supervisors, five masons and seven assistant masons employed by a private construction company. The two on-site supervisors were foreign to Mozambique and had limited Portuguese language proficiency. They could communicate with their Mozambican employees, but not with the trainer; thus, the supervisors did not participate in the training.

### 3.2. Evaluation of Control Guidance

The walkthrough survey guide included a description of the good work practice categories presented on the task-based control guidance sheets, worksite description, equipment, tools and materials used. The assessment tool was a checklist showing the identified good work practices and with open space to add any additional work practices observed [2,14]. Comparison of the recorded observations by one of the authors (CM) with the observers during random checks showed complete agreement during all three activities—pretraining, training and posttraining. For data analysis, only the recordings made by the observers were used.

Pretraining work practice observations: Crew 1 worked on two sites before the training. Tools used prior to the training were: sledge hammers, chisels, scrapers, shovels, buckets, pickup truck, wheel barrow and their hands to remove debris. Tools were selected for the task as the work progressed. First, the area to be demolished was marked with a pencil and then the crew used hand tools for demolition, followed by breaking up with a sledge hammer. The cleaning and removal task was conducted to concentrate the debris in one area and the debris was transferred to a container and carried to a disposal or salvage point. Potable water and toilet facilitates were the only good work practice components accessible to workers; however they did not procure and use water to suppress dust.

Crew 2 worked on one renovation project which involved some demolition operations before and after training. The crew used the following tools: sledge hammers, chisels, scrapers, circular saw and shovel. They used their hands, push broom, and buckets for debris removal. Tools were selected for the task as the work progressed. This crew worked in teams. One team marked the area with white chalk and used a circular saw to initiate the demolition; another team was assigned to breaking up the concrete and masonry structure with chisel and hammer or sledge hammer, and a third team worked on cleaning and removing the debris to a designated point. Some employees had a military uniform and hard hat, and wore surgical masks on a voluntary basis. Also, potable water and toilet facilities were available, but were not discussed as a source of water to reduce dust exposures. 

The measure of effectiveness of the intervention was integrated within the worker training evaluation using a seven-level evaluation model. The results for each level are summarized in Table 1.

**Table 1:** Summary results of worker training evaluation using seven levels framework.

#	Evaluation level	Results for each level
I	Attendance	25 workers achieved 100% and one achieved 97.6% of attendance.
II	Formative evaluation	Stakeholders in U.S. and Mozambique provided input and support for the development, implementation and evaluation of checklists.
III	Satisfaction and opinion of the trainees	54% of all participants, provided feedback *. Thirteen of 14 (93%) trainees were satisfied. 93% of trainees rated the training and the work practice components in the checklist to be “useful” and “effective”. Of the controls presented, water to suppress was rated highest, as extremely useful or useful by 71 and 21% of trainees respectively. Only one trainee considered training difficult. 36% of trainees considered the facilitator not flexible with the agenda. All trainees proving written feedback indicated the need for regular training for themselves and other workers exposed to dust.
IV	Knowledge acquisition	12% of trainees indicated knowledge before training and 100% of trainees indicated knowledge of silica dust exposure prevention and controls after the training. The difference of proportion was statistically significant (*p* < 0.01).
V	Skills acquisition	Two participants missed items during preplanning tasks and one participant missed an item during the check after task completion. After reexplanation and demonstration all the steps were successfully completed. Trainees mastered the use of task-based good work practice guidance sheets. Observers and investigator results were similar and consistent.
VI	Transfer of learning to the workplace	Pretraining: work practice components used: 8%. Posttraining: Work practice components used: 63%.
VII	The impact of the training	Work practice components used: 67 to 71% units of change after training. The difference of proportions pre-and-post training was statistically significant (*p* < 0.05).

* Fourteen of the 26 participants (54%) provided written feedback on the training evaluation instruments. This represents 100% of Crew 1. The Crew 2 feedback was not accomplished because of communication difficulties with supervisors and limited time.

The results of observed work practices before and after training are shown below in Table 2.

**Table 2:** List of components of the good work practice control guidance observed before and after training of demolition workers (–: if not used and +: if used).

#	Components of good work practice control guidance sheets	Timing of Observations	
		Before	After
**1**	Preplanning	–	+
	Displaying warning signs, checking if all tools and supplies are available and functioning well	–	+
**2**	Water for dust suppression	–	+
	Water hose spray	–	+
	Sprinkling by hand or other appropriate resources	–	+
	Wet wiping	–	+
**3**	High efficiency particulate air (HEPA) filter vacuum	–	–
**4**	Natural ventilation		
	Free air circulation (doors and windows open if possible)	–	+
	Working upwind/upstream of the dust‑generating source	–	+
**5**	Basic personal protective equipment (PPE)		
	Safety glasses with side shields	–	+
	Hard hat	–	+
	Safety shoes (boots or steel toe shoes)	–	–
	Work gloves	–	+
	Hearing protection	–	+
	Long sleeves and long pants	–	–
**6**	Personal hygiene practices		
	Hand washing facilities	–	+
	Water for cleaning tools and PPE	–	+
	Separated space for eating and drinking	–	+
**7**	Basic sanitation practice		
	Potable water	+	+
	Toilet	+	+
**8**	Self post-performance evaluation	–	+
**9**	Other control measures		
	Containment or isolation	–	–
	Local exhaust ventilation	–	–
	General mechanical ventilation	–	–
	Respiratory protection equipment (N95 mask)	–	+

Work practices were also documented in photographs obtained throughout the intervention; selected photos are shown below.

**Figure 3:**
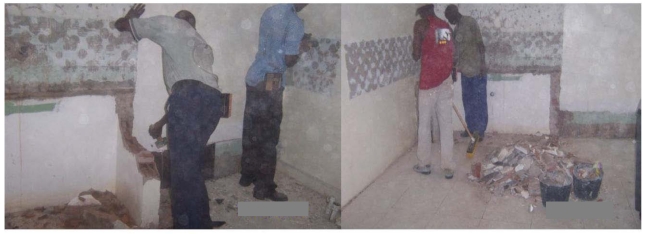
Photo of workers from Crew 1 breaking up masonry structure and clean-up demolition debris before worker training (Photographer: C.M.).

**Figure 4:**
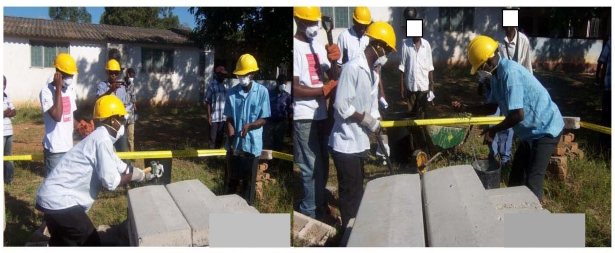
Photo of a demolition crew (employees and supervisors) during the simulation exercise (Photographer: C.M.).

**Figure 5:**
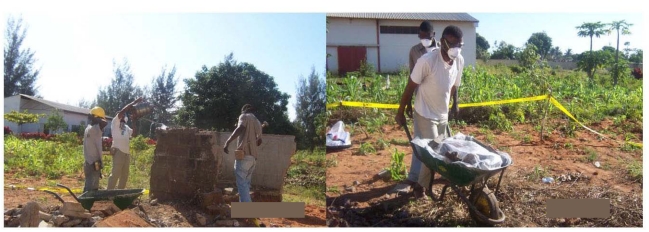
Photo of workers (one mason and two assistant masons) sprinkling water before breaking up concrete/masonry structure and pushing a wheelbarrow filled and covered with plastic sheet (after the training to use checklist) (Photographer: C.M.).

## 4. Discussion

Our previous work identified small-scale demolition of concrete and masonry structures/buildings to be dusty and that workers were likely at risk of silica exposure [18]. These operations are conducted for short durations at temporary work sites. As many types of engineering controls in guidance used in developed countries generally are not available in Mozambique, the development and evaluation of alternative control guidance was undertaken. The work described here provides a framework for the development of regionally-appropriate control guidance checklists. This work provided empirical data on the adaptation of task-based control guidance checklists for three tasks. A fourth checklist developed initially was found to cover a task not routinely done and was dropped. This work organization was reported by the supervisors and verified during walkthroughs at the site. It is not known if this combination of tasks is unique to the crews participating in this work, or is now the more usual work organization [14,15,16,17,18]. The process described allowed for timely modification of checklists, consistent with the work practices used at the time of training.

A one-page checklist was created for employees and multiple-page checklist was created for supervisors for three small-scale demolition tasks; both English and Portuguese versions were developed. The checklist format presented is much shorter than other tools [15,16] due to narrowly defining each task and a focus on work practices rather than engineering controls that are not available locally. These guidance sheets were produced with the input from workers, management and occupational hygiene practitioners. For more complex work locations, a range of task-specific sheets could be created, and the training program developed to include the selection of relevant tasks by the participants. The full complement of sheets would be available, and could be a resource for the participants at future work sites, where work organization might vary.

The work practices at each task were evaluated using a dichotomous variable (yes/no). While the answer “yes” was verification that the work practice was being followed, the answer “no” indicated a need for remediation before continuing with the task demonstration. The multiple-page supervisor guidance sheets included the one-page employee checklist, plus information on the rationale for silica dust exposure concern (regulatory compliance, costs, and health effects), exposure prevention and control, supervision and training, resources and links to further and useful information. These supervisor guidance checklists may be a resource for future site-specific good work practice control strategies, supervisor guidance, employee training to reinforce use of good work practices and as toolbox talks. Compared with other good work practice guidance sheets these checklists are specific for employees and supervisors and written in the appropriate language. The recommended exposure controls included in the training were identified using input from all stakeholders and were available locally. 

The systematic and iterative logic framework used to develop these checklists (see Figure 1) is consistent with the framework presented by Hales *et al.* [26] in the development of medical checklist for improved quality of patient care and the logic and methodology of checklist development and evaluation presented by Stufflebeam [19]. This process of checklist development can be used for new work activities or to adapt checklists from existing models.

Worker training was identified as the best strategy to facilitate use of the control guidance checklists. Training development and delivery was based on cognitive theory and integrated technical information into interactive and hands-on sessions consistent with effective worker training [27] and transfer of learning the workplace [28]. The checklist and training program were prepared in English, translated and delivered in Portuguese, incorporating linguistic and cultural considerations for the population to be trained [29]. Quality assurance for the translation included review and back-translation into English. 

The seven-level evaluation framework provided more specific criteria than the widely used, four-level evaluation model developed by Kirkpatrick [30]. The design and intervention evaluation methods documented a short-term effect of the training on work practices. Twenty-two employees and four supervisors attended the interactive, hands-on worker training. Ninety-six percent of participants achieved perfect attendance. One supervisor (4%) did not complete the three posttraining items. The attendance counting results were equal for one author (CM) and two data collectors. This level of attendance indicated a high level of interest and commitment from participants. The formative evaluation of the checklist and training material development performed both by the US professionals and workers, and on-site supervisors and employees helped assure relevance of the instruments [26]. 

Fourteen of the 26 participants (54%) provided some feedback on the training evaluation instruments. A language barrier and short period of time available for this aspect of data collection prevented other crew members from providing input. Among the supervisors, two were immigrants who did not read Portuguese and did not participate in the training. Unfortunately time did not allow further accommodation of contractor management personnel, such as hiring a translator to help assure employees had time to complete feedback evaluations. Time to deal with special training alternatives should be built into the program plan. 

Knowledge gain was documented by participants raising their hands to indicate knowledge about various aspects of silica hazards and controls before and after training. The assumption that any worker would not raise his hand without knowledge about the topic could be an error; however, those who raised a hand were asked to provide information about their knowledge and this was recorded on a flipchart. These responses were generally consistent with knowledge. Those who did not raise their hand could have been knowledgeable, and therefore the data reported could be an underestimate. Although the knowledge gain was assessed using this crude measure it was consistent with the participatory approach [26,27,28,29,30]. Alternative knowledge gain assessment methods are the use of a pre- and post-test written items or retrospective pretest [31]. These methods may be integrated into the initial training or used at refresher training.

A strength of the program evaluation is that 9 of 14 trainees provided anonymous open-ended immediate comments. These included findings that the checklist and training program were useful to understanding the health hazards and how to reduce silica dust exposures during their work, and the need for more opportunities for training including refresher training and an extension to other workers exposed to dust. Participants wrote their concern regarding lack of schedule flexibility and lack of ability to accommodate unplanned or unexpected events. Lack of understanding of the material was raised by two respondents. These results show opportunity for training improvement, especially the need to allocate work time for training and not be restricted to using lunch breaks and the time before work. Time to discuss the feedback with participants would be useful as part of program improvement. 

The skills performance observations indicated that all the checklist actions were successfully completed during the simulation by all 26 participants, although some required remedial discussions and demonstrations for full mastery were necessary. The observations and completion of the training performance checklist were consistent for both the investigator and the data collectors; therefore, we did not evaluate differences or trends over time. The documented changes are evidence of motivation to implement good work practices and use available tools; for example, the workers found sources of water in order to utilize wet methods and demonstrated awareness for the use of natural ventilation to reduce exposure. Effort is needed to increase availability of HEPA vacuum equipment for use in demolition activities. 

Longer follow-up is needed to determine if changes in work practices can be sustained by the crew members and supervisors at this location, and as they move to other work sites. Refresher training may also be needed, if the implementation of good work practices is documented to decline over time.

Training transfer generally refers to the use of new knowledge and skills on the job. For transfer to occur, learned behavior must be generalized to the job context and maintained over a period of time [29,30,31]. Substantial differences were noted in comparing before and after training work practices including, improved basic sanitation, executed demolition plan that included the use of wet methods to suppress dust, natural ventilation by opening windows and doors, and identified the airflow/wind direction in order to be positioned on the upstream or upwind side of the dust generation source, used available personal protective equipment, used wet methods to clean their PPE and tools, and post-work assessment. Respiratory protection is generally not available, but was provided as part of the data collection plan.

A significant change in work practice components was shown immediately after the training intervention, indicating transfer of learning from the interactive session to the workplace. The two demolition crews worked in different locations, demolishing dissimilar structures; however, the work practices were similar in both throughout the sites and between crew members doing the same task. All employees appeared highly motivated to participate in the program and use the checklists. In summary, results indicated that trainees understood the relationship between training content and work tasks and transferred knowledge and skills to the work setting.

Continuation of this transfer is dependent on the ability to access water and reinforcement factors from the established organization and administration to take time for planning and work organization. With this structure in place, an opportunity is available to the trained workers to use the knowledge and skills, discuss work practices, provide feedback and recommendations, and share their new knowledge and skills with others. This training was not requested by the management or employees but was proposed by the investigator; as designed, the workers, management and supervisors must oversee continued use of good work practices. Follow-up is required to investigate if this has occurred. 

Another way of investigating the effectiveness of the work practice changes would be pre- and post-training measurement of silica dust exposure. This strategy was implemented, but did not produce meaningful results because demolition tasks are completed in a short period of time (5–120 min) and analyses for silica were below the limit of detection. However, respirable dust mass was reduced 40 to 60% after the training intervention, consistent with reductions reported in other intervention studies of similar tasks, tools and materials [1,2,3].

This pilot study included development and evaluation of an alternative to engineering controls recommended in control guidance sheets in use in industrialized countries, since these approaches are often not available in developing nations, especially at small-scale work such as demolition operations. Using a training intervention, guidance and short checklists were used by employees to improve work practices. The study also showed the innovation of the workers in identifying water resources in order to use wet methods to suppress dust. Although the work reported here focuses on small-scale demolition operations in Mozambique, the concepts and principles may be relevant for other dust generating construction activities, especially in small operations. Training and checklists will help workers recognize the hazard of silica dust, identify controls before starting work and encourage implementing controls during each task. This work provides a framework for adaptation of the existing good work practice control guidance and control banding toolkits developed in industrialized countries to developing countries.

## 5. Conclusions

A step-wise approach to adapting checklists and guidance for small-scale demolition in a developing country is described. Use of the checklists was implemented by trained workers and found in the short-term to improve work practices that would reduce exposures to dust. Following training, substantial changes in work practices were observed, including preplanning, use of wet methods, natural ventilation and end-of-task review. Consistent ratings by the trained observers suggest good reliability and validity of the walkthrough survey guidance and the observation data instruments. Additional follow-up is needed to determine the long-term impact on sustained changes in work practices, and may aid efforts to evaluate the need for refresher training. Replication with other demolition workers and other small-scale work activities is needed to provide insights regarding generalizability of the approach described. 
